# A preliminary study for constructing a bioartificial liver device with induced pluripotent stem cell-derived hepatocytes

**DOI:** 10.1186/1475-925X-11-93

**Published:** 2012-12-07

**Authors:** Masaya Iwamuro, Hidenori Shiraha, Shuhei Nakaji, Masumi Furutani, Naoya Kobayashi, Akinobu Takaki, Kazuhide Yamamoto

**Affiliations:** 1 Department of Gastroenterology and Hepatology, Okayama University Graduate School of Medicine, Dentistry, and Pharmaceutical Sciences, 2-5-1 Shikata-cho, Okayama, Okayama, 700-8558, Japan; 2 Department of Biomedical Engineering, Okayama University of Science, Okayama, 700-0005, Japan; 3 Central Research Laboratory, Okayama University Medical School, Okayama, Okayama, 700-8558, Japan; 4 Department of Surgery, Okayama Saidaiji Hospital, Okayama, Okayama, 704-8192, Japan

**Keywords:** Induced pluripotent stem cells, Bioartificial liver system, Hepatocyte differentiation, Hollow fiber bioreactor, Antibody-mediated rejection

## Abstract

**Background:**

Bioartificial liver systems, designed to support patients with liver failure, are composed of bioreactors and functional hepatocytes. Immunological rejection of the embedded hepatocytes by the host immune system is a serious concern that crucially degrades the performance of the device. Induced pluripotent stem (iPS) cells are considered a desirable source for bioartificial liver systems, because patient-derived iPS cells are free from immunological rejection. The purpose of this paper was to test the feasibility of a bioartificial liver system with iPS cell-derived hepatocyte-like cells.

**Methods:**

Mouse iPS cells were differentiated into hepatocyte-like cells by a multi-step differentiation protocol via embryoid bodies and definitive endoderm. Differentiation of iPS cells was evaluated by morphology, PCR assay, and functional assays. iPS cell-derived hepatocyte-like cells were cultured in a bioreactor module with a pore size of 0.2 μm for 7 days. The amount of albumin secreted into the circulating medium was analyzed by ELISA. Additionally, after a 7-day culture in a bioreactor module, cells were observed by a scanning electron microscope.

**Results:**

At the final stage of the differentiation program, iPS cells changed their morphology to a polygonal shape with two nucleoli and enriched cytoplasmic granules. Transmission electron microscope analysis revealed their polygonal shape, glycogen deposition in the cytoplasm, microvilli on their surfaces, and a duct-like arrangement. PCR analysis showed increased expression of albumin mRNA over the course of the differentiation program. Albumin and urea production was also observed. iPS-Heps culture in bioreactor modules showed the accumulation of albumin in the medium for up to 7 days. Scanning electron microscopy revealed the attachment of cell clusters to the hollow fibers of the module. These results indicated that iPS cells were differentiated into hepatocyte-like cells after culture for 7 days in a bioreactor module with a pore size of 0.2 μm.

**Conclusion:**

We consider the combination of a bioreactor module with a 0.2-μm pore membrane and embedded hepatocytes differentiated from iPS cells to be a promising option for bioartificial liver systems. This paper provides the basic concept and preliminary data for an iPS cell-oriented bioartificial liver system.

PACS code: 87. Biological and medical physics, 87.85.-d Biomedical engineering, 87.85.Lf Tissue engineering, 87.85.Tu Modeling biomedical systems.

## Background

Non-biological artificial liver systems are external supportive devices that are currently applied to patients with liver dysfunction. The basic mechanism of a non-biological artificial liver system is albumin dialysis, and the two representative devices include extracorporeal albumin dialysis (MARS®) and fractionated plasma separation and absorption (Prometheus®). Improvement of jaundice, hepatic encephalopathy, systemic hemodynamic dysfunction and/or portal hypertension has been observed by these systems [1,2]. However, these outcomes are temporary, and randomized control trials have failed to show clear a benefit of such systems to the long-term survival of these patients [3-7]. In theory, an ideal artificial liver system could permanently replace liver functions, providing most or even all normal liver functions such as detoxification and synthesis [8,9]. To achieve replacement of whole liver function, investigators have tried to develop cell-based external artificial biological devices, namely, a bioartificial liver (BAL) system.

A bioartificial liver (BAL) system is composed of bioreactors and embedded hepatocytes that provide protein synthesis, ureogenesis, ureagenesis and glucogenesis, and detoxification through P450 activity [8,10,11]. Various cell types, such as primary human hepatocytes, immortalized cell lines of C3A human hepatoblastoma cells, and cryopreserved porcine hepatocytes have been studied for BAL applications. Several studies of BAL use have been reported as clinical trials [12-15], although none of these BAL systems has yet been approved by the FDA. Major problems of BAL systems include insufficient supply of functional hepatocytes, premature death of hepatocytes by immune mechanisms, and the inadequate performance of devices for mass transfer [16].

Induced pluripotent stem (iPS) cells are artificially derived stem cells that have been genetically reprogrammed to an embryonic stem cell-like state. iPS cells were first created in 2006 from mouse fibroblasts and in 2007 from human fibroblasts by forced expression of specific genes [17-19]. iPS cells are pluripotent and are able to proliferate unlimitedly *in vitro*. This technical breakthrough in creating iPS cells from somatic cells has noteworthy implications for producing large amounts of iPS cell-derived functional hepatocytes that could overcome immunological rejection. In this context, iPS cells are expected as one of the favorable cell sources for cell therapy. The purpose of this paper is to test the feasibility of a BAL system with iPS cell-derived hepatocytes. The optimal pore size for BAL devices is also investigated.

## Methods

### Culture of undifferentiated mouse iPS Cells

Mouse iPS cells were purchased from Riken Cell Bank (Cell No. APS0001, Cell name iPS-MEF-Ng-20D-17, Lot No. 006) [20]. Mouse iPS cells of 11–20 passages were maintained by culture on a feeder layer of mouse embryo fibroblasts (MEF) (Dainippon Pharmaceutical, Osaka, Japan) inactivated by mitomycin C (Biomol, Enzo Life Sciences International, PA) on gelatin (Specialty Media, Chemicon International, MA)-coated plates. iPS cells were cultured with Complete ES cell medium (Specialty Media, Chemicon International) supplemented with 1000 U/ml recombinant leukemia inhibitory factor (LIF) (ESGRO, Chemicon International) at 37°C in 5% CO_2_. Each passage was carried out before cells reached confluency [21].

### In vitro differentiation of hepatocyte-like cells from undifferentiated iPS cells

Differentiation was carried out in four stages as described previously, with minor modification [22].

Stage 1: Formation of Embryoid Bodies (Days 0–1). iPS cells growing on feeder cells were dispersed by treatment with trypsin-EDTA (Sigma-Aldrich Japan, Tokyo, Japan) and collected by centrifugation at 800 rpm for 3 min. Cells were then resuspended in Knockout DMEM (Gibco, Invitrogen, CA) supplemented with 15% knockout serum replacement (KSR) (Gibco), 1% nonessential amino acids (MP Biomedicals, CA), 1% 2-mercaptoethanol (Gibco), 1% penicillin/streptomycin (Sigma-Aldrich Japan), and 1% l-glutamic acid (DS Pharma Biomedical, Osaka, Japan). The iPS cells were then transferred to ultra-low-attachment six-well plates (Corning, NY) and were cultured free floating in the culture medium at a density of 2 × 10^5^ cells/2 ml/well to induce the formation of embryoid bodies (at day 0). Half of the medium was replaced daily.

Stage 2: Induction of Definitive Endoderm (Days 2–4). At day 2, embryoid bodies were collected from the single well and centrifuged at 1000 rpm for 3 min. Embryoid bodies were then resuspended in 9 ml of Knockout DMEM, 1% penicillin/streptomycin, 1% l-glutamic acid, 100 ng/ml activin A (R&D Systems, MN), and 100 ng/ml basic fibroblast growth factor (FGF) (Peprotech, NJ) to induce a definitive endoderm. In each well of a gelatin-coated 12-well plate, 1.5 ml of the medium containing embryoid bodies was seeded. Embryoid bodies were cultured for an additional 3 days. KSR was supplemented at days 3–4 and the concentrations were varied: 0.2% for day 3 and 2.0% for day 4. Half of the medium was replaced daily.

>Stage 3: Differentiation of Premature Hepatocyte-Like Cells (Days 5–11). At day 5, the medium was replaced by Knockout DMEM supplemented with 1% KSR, 1% nonessential amino acids, 1% l-glutamic acid, 1% dimethyl sulfoxide (DMSO) (Sigma-Aldrich, Irvine, UK), and 100 ng/ml hepatocyte growth factor (HGF) (Peprotech, NJ) to induce premature hepatocyte-like cells. The medium was replaced every two days.

Stage 4: Differentiation of Matured Hepatocyte-Like Cells (Days 12–18). At day 12, the medium was replaced by Knockout DMEM supplemented with 1% KSR, 1% nonessential amino acids, 1% l-glutamic acid, and 40 ng/ml dexamethasone (Sigma-Aldrich) to induce hepatocyte-like cells (iPS-Heps). The medium was replaced every two days. The differentiation program was finished at day 19.

### Characterization of differentiated hepatocyte-like cells by imaging modalities

Phase-contrast images were observed by CKX41 phase-contrast microscopy (Olympus, Tokyo, Japan). Periodic acid Schiff (PAS) analysis was performed with PAS staining solution (Muto Pure Chemicals, Tokyo, Japan), according to the manufacturer’s instructions. Briefly, the cultured cells were fixed in 3.3% formalin for 10 min, and intracellular glycogen was stained using a PAS staining solution. For transmission electron microscope analysis, samples were fixed using 2% paraformaldehyde and 2% glutaraldehyde in 0.1 mol/L phosphate buffer solution at 4°C for 18 h. The samples were then postfixed with 2% OsO_4_ and dehydrated by ethanol before being embedded in epoxy resin. Ultra-thin sections were examined under an H-7100 transmission electron microscope (Hitachi, Tokyo, Japan).

### Isolation of RNA and Reverse-transcription polymerase chain reaction

Reverse-transcription polymerase chain reaction (RT-PCR) was carried out at various points of the differentiation program to determine the degree to which iPS cells differentiated toward iPS-Heps. RNA was extracted using an RNeasy Mini Kit (QIAGEN, Duesseldorf, Germany) according to the manufacturer’s instructions. Complementary DNA was prepared using MuLV reverse transcriptase (Applied Biosystems, CA) and RNase inhibitor (Applied Biosystems) from 2 μg of total RNA. RT-PCR was performed with TaKaRa LA Taq DNA polymerase (Takara Bio Inc., Otsu, Japan). Primers used for RT-PCR are listed in Table 1. PCR products were resolved on 2% agarose gels and visualized by ethidium bromide staining. Real-time PCR was performed on a LightCycler 1.5 Instrument (Roche Applied Science, IN) with LightCycler FastStart DNA master SYBR green I (Roche Applied Science, IN) according to the manufacturer’s instructions. Primers are listed in Table 2.

**Table 1:** RT-PCR primers used in the present study

**Gene**	**Forward Primer**	**Reverse Primer**	**Product Size (bp)**
AFP	CACTGCTGCAACTCTTCGTA	CTTTGGACCCTCTTCTGTGA	300
Alb	GACAAGGAAAGCTGCCTGAC	TTCTGCAAAGTCAGCATTGG	174
Trf	ACCATGTTGTGGTCTCACGA	ACAGAAGGTCCTTGGTGGTG	135
CPS	AGGATGTCAAGGTGTTTGGC	GCTTAACTAGCAGGCGGATG	93
β-actin	GACCTCTATGCCAACACAGT	AGTACTTGCGCTCAGGAGGA	139

*AFP*, α-fetoprotein; *Alb*, Albumin; *Trf*, transferrin; *CPS*, carbamoyl phosphate synthetase.

**Table 2:** Real-Time RT-PCR primers used in the present study

**Gene**	**Forward Primer**	**Reverse Primer**	**Product Size (bp)**
AFP	AAACATCCCACTTCCAGCAC	AGCGAAATGTAGCAGGAGGA	94
Alb	GTTCGCTACACCCAGAAAGC	CCACACAAGGCAGTCTCTGA	124
Trf	ACCATGTTGTGGTCTCACGA	ACAGAAGGTCCTTGGTGGTG	135
CPS	AGGATGTCAAGGTGTTTGGC	GCTTAACTAGCAGGCGGATG	93
β-actin	GACCTCTATGCCAACACAGT	AGTACTTGCGCTCAGGAGGA	139

*AFP*, α-fetoprotein; *Alb*, Albumin; *Trf*, transferrin; *CPS*, carbamoyl phosphate synthetase.

### Hepatocyte isolation

Normal primary hepatocytes were prepared as positive controls for PCR analysis and ELISA assay. Mouse hepatocytes were isolated from the whole liver of an adult C57BL/6 mouse (male, 8–12 weeks old) by the two-step liver perfusion method [23] with the commercially available liver perfusion medium, liver digest medium, and hepatocyte wash medium (Life Technologies, CA). Cell viability was determined by trypan blue exclusion, and cells obtained with more than 60% viability were plated on collagen-I coated dishes (Iwaki, Tokyo, Japan). Isolated hepatocytes were maintained with HepatoZYME-SFM medium (Life Technologies) for 24 hours, and then the cells and the medium were collected.

### Measurement of albumin production by ELISA

The supernatant of the mouse iPS cell culture was collected. The amount of albumin secreted into the culture medium was measured by an albumin enzyme-linked-immunosorbent assay (ELISA) kit (Immunology Consultants Laboratory, OR), as per the manufacturer’s instructions.

### Evaluation of permeability of albumin and IgG in bioreactor modules with different pore sizes

To test the permeability of albumin and IgG in bioreactor modules, a CellMax hollow fiber bioreactor system (Spectrum Laboratories Inc., CA) and a polysulfone membrane cartridge with 145 cm^2^-surface area and pore sizes of 100 kilodaltons, 500 kilodaltons, or 0.2 μm (MidiKros Plus; Spectrum Laboratories Inc., CA) were used. The intracapillary space of the cartridge was perfused with 200 ml of perfusion medium at the slowest flow rate of the system. The perfusion medium consisted of Knockout DMEM supplemented with 1% KSR, 1% nonessential amino acids, 1% l-glutamic acid, and 40 ng/ml dexamethasone. After the injection of 20 μg of mouse albumin or 2 μg of mouse IgG resuspended with 5 mL of perfusion medium into the extracapillary space, the drained medium from the intracapillary space was collected at 0, 30, 60, 90, 120, 150, and 180 min. The amount of permeated albumin and IgG was measured by a mouse albumin ELISA kit and mouse IgG ELISA kit (Immunology Consultants Laboratory), according to the manufacturer’s instructions.

### Culture of iPS-Heps and isolated hepatocytes in a bioreactor module

The culture of iPS-Heps in a bioreactor module was carried out by a CellMax hollow fiber bioreactor system and a polysulfone membrane cartridge with a 145-cm^2^ surface area and 0.2-μm pores. Prior to the injection of iPS-derived premature hepatocyte-like cells, the system was perfused with 200 ml of perfusion medium as described above. Cells at the end of stage 3 (day 12) of differentiation were collected from two 12-well plates and centrifuged at 1000 rpm for 3 min. Cells were then resuspended in 2 ml of perfusion medium and were injected into the extracapillary space of the bioreactor module. Cells were incubated at 37°C in 5% CO_2_ for up to 7 days at the slowest flow rate of the system. The medium was not replaced during the period of incubation. The culture of isolated mouse hepatocytes was performed by the same system with 5.0 × 10^5^ hepatocytes with 200 ml of HepatoZYME-SFM medium.

To monitor the amount of albumin secreted into the intracapillary space, medium was collected every 24 hours. The concentration of urea was also measured by SRL, Inc. (Tokyo, Japan) after the 7-day culture.

### Scanning electron microscope analysis

After the 7-day culture of iPS-Heps in a bioreactor module, the cartridge was dismantled and hollow fibers were brought out. The hollow fibers were immersed for 8 h in a mixture of 2% paraformaldehyde and 2% glutaraldehyde in 0.1 mol/L phosphate buffer solution (pH 7.4). The samples were washed twice in phosphate buffer solution for 1 h each and postfixed for 2 h in 1% OsO_4_ in phosphate buffer. Samples were rinsed for 1 h in phosphate buffer and dehydrated in an ascending ethanol series (50, 70, 90, 95, and 99.5%; 20 min each) before 1 h of drying with tert-butyl alcohol. The polished surface coated with osmium (HPC-1S type osmium coater; Shinku Device Co. Ltd., Ibaraki, Japan) was examined closely with an S4800 scanning electron microscope (Hitachi).

## Results

**Figure 1:**
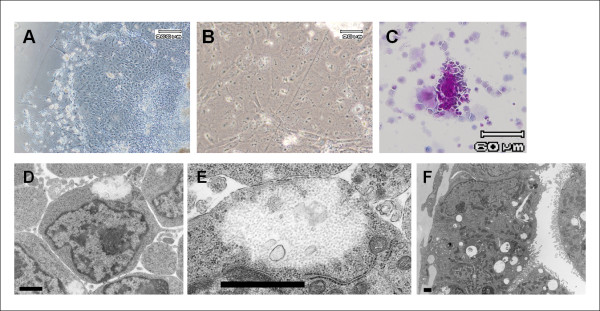
**Morphological evaluation of differentiated iPS cells.** Differentiated iPS cells (day 19) had a polygonal shape with two nucleoli and enriched cytoplasmic granules (**A**,**B**). PAS staining demonstrated intracellular deposition of glycogen (**C**). Transmission electron microscopy revealed their polygonal shape and glycogen deposition in the cytoplasm (**D**,**E**). Differentiated cells partly formed duct-like structures with microvilli on the cell surface which projected into the intercellular space (**F**). These morphological characteristics resembled those of normal mouse hepatocytes in culture. Scale bar = 1 μm.

After 19 days of the differentiation program, the cells had a polygonal shape with two nucleoli and enriched cytoplasmic granules (Figure 1A,B). Glycogen storage was confirmed by PAS staining (Figure 1C). Transmission electron microscope analysis also demonstrated that the polygonal cells had glycogen deposition in the cytoplasm, namely, glycogen fields (Figure 1D,E). Differentiated cells partly formed duct-like structures, and the cells had many microvilli projecting into the intercellular space (Figure 1F). These morphological characteristics were compatible with those of normal mouse hepatocytes in culture.

**Figure 2:**
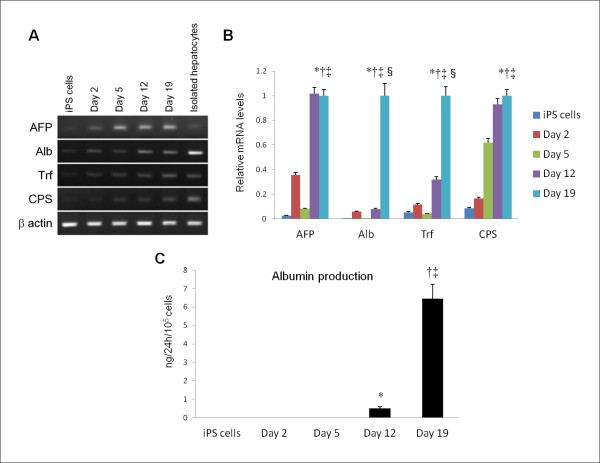
**Functional analysis of iPS cell-derived hepatocyte-like cells.** RT-PCR analysis showed progressive increase of mRNA expression of hepatocyte-enriched markers, such as albumin, the urea cycle enzyme carbamoyl phosphate synthetase, and transferrin over the course of differentiation program (**A**,**B**). However, gene expression levels of such markers in differentiated iPS cells (day 19) were much lower than those of isolated hepatocytes. Moreover, strong expression of α-fetoprotein at day 19 indicated that the final product of the differentiation program was not fully matured hepatocytes. Real-time PCR analysis of gene expression levels relative to β actin was performed using three independent experiments. *p < 0.01 iPS cells vs differentiated cells (day 19). †p < 0.01 differentiated cells (day 2) vs differentiated cells (day 19). ‡p < 0.01 differentiated cells (day 5) vs differentiated cells (day 19). §p < 0.01 differentiated cells (day 12) vs differentiated cells (day 19). An ELISA assay showed that the amount of albumin produced by differentiated cells (day 19) was 6.45 ± 0.78 ng/24h/10^5^ cells (**C**). *p < 0.01 iPS cells vs differentiated cells (day 12). †p < 0.01 iPS cells vs differentiated cells (day 19). ‡p < 0.01 differentiated cells (day 12) vs differentiated cells (day 19). The ELISA data shown are means ± SDs from three independent experiments.

RT-PCR analysis showed that the expression of albumin, a marker for mature hepatocytes, progressively increased over the course of the differentiation program (Figure 2A,B). Albumin production in the culture media was also observed by ELISA assay. At day 19, the amount of albumin produced by differentiated cells was 6.45 ± 0.78 ng/24h/10^5^ cells (Figure 2C). Gene expression levels of hepatocyte-enriched markers, such as the urea cycle enzyme carbamoyl phosphate synthetase and transferrin also intensified in a time-dependent manner. The result confirmed progressive differentiation toward hepatocytes in the population of cells. However, the expression level of iPS-Heps was much lower than that of isolated hepatocytes. Moreover, α-fetoprotein expression, which is present in the endoderm but is not expressed by mature hepatocytes, was still detected after 19 days of differentiation. These gene expression profiles indicate that the final products of our differentiation program were not fully matured hepatocytes.

**Figure 3:**
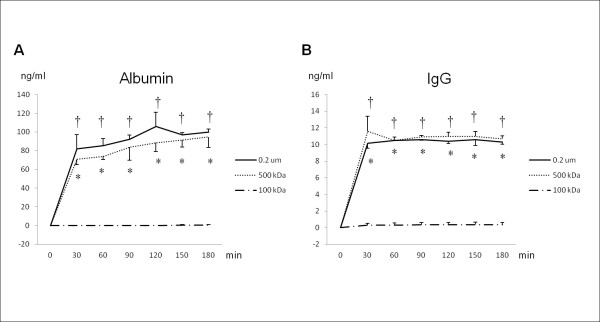
**Result of permeability assay of membranes with three different pore sizes.** The permeability assay in this study revealed that neither albumin nor IgG could pass through a membrane with 100-kilodalton pores, whereas both albumin and IgG permeated membranes with 500-kilodalton pores and 0.2-μm pores. None of the three membranes (100-kilodalton, 500-kilodalton, or 0.2-μm pores) could separate albumin and IgG. *p < 0.01 membranes with 500-kilodalton pores vs 100-kilodalton pores. †p < 0.01 membranes with 0.2-μm pores vs 100-kilodalton pores. The data shown are means ± SDs from three independent experiments.

Albumin is the main protein of blood plasma and is produced in the liver. Albumin serves as a carrier for molecules of low water solubility, including several toxic substances that accumulate in liver failure (e.g., bilirubin) and some drugs (e.g., warfarin, phenylbutazone, clofibrate, and phenytoin). On the other hand, IgG is one of the immunoglobulins that initiate antibody-mediated immune responses. Accordingly, a membrane that permits albumin permeation and blocks IgG permeation is favorable for a BAL device. Theoretically, a pore membrane of 100 kilodaltons would satisfy this condition, because the pore size is larger than the molecular weight of albumin (67 kilodaltons) and is smaller than IgG (147 kilodaltons). However, the permeability assay in this study revealed that neither albumin nor IgG could pass though membrane with 100-kilodalton pores, whereas both albumin and IgG permeated cartridges of 500-kilodalton pores or 0.2-μm pores (Figure 3). These results were in concordance with those reported by other authors [24]. Consequently, it does not seem feasible to separate albumin and IgG by filtration with a specific pore size. Based on this reasoning, we concluded that it is impossible to construct the BAL system with isolated hepatocytes that have both a sufficient supply of proteins and an immunological barrier. In this context, iPS cell-derived hepatocytes are the most adequate cell source for constructing such ideal BALs.

**Figure 4:**
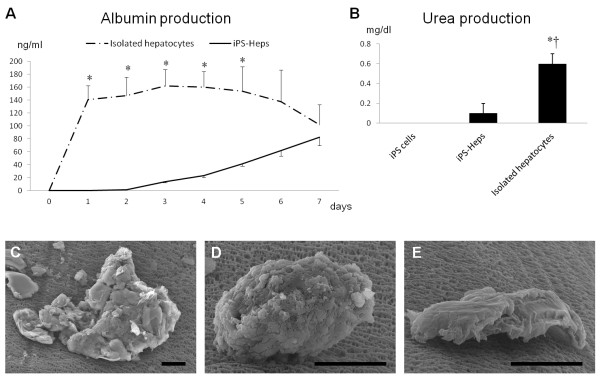
**Culture of iPS-Heps in bioreactor modules.** An ELISA assay showed a linear increase in albumin for 7 days in bioreactor modules embedding iPS-Heps (**A**). The amount of albumin produced by iPS-Heps in the bioreactor module at day 7 was 82.4 ± 12.7 ng/ml. Isolated hepatocytes in the bioreactor modules produced a significant amount of albumin on day 1 (141.1 ± 21.3 ng/ml) The amount had only slightly increased by day 3, then the albumin concentration sequentially decreased over the following 4 days. The ELISA data shown are means ± SDs from three independent experiments. *p < 0.01 iPS cells vs isolated hepatocytes. iPS-Heps produced urea at the concentration of 0.1 ± 0.1 mg/dl, whereas hepatocytes produced 0.6 ± 0.1 mg/dl (**B**). *p < 0.01 iPS cells vs isolated hepatocytes. †p < 0.01 iPS-Heps vs isolated hepatocytes. Scanning electron microscopy analysis showed cell clusters attached to the extracapillary membrane (**C**,**D**,**E**). These results indicated that iPS-Heps existed in the extracapillary space in an adhesive state, and those cells survived for up to 7 days of culture. Scale bar = 10 μm.

The ELISA assay of iPS-Heps culture in the bioreactor modules showed accumulation of produced albumin in the medium over the culture period (Figure 4A). Ultimately, the production of albumin by iPS-Heps was 82.4 ± 12.7 ng/ml. On the other hand, isolated hepatocytes in the bioreactor modules produced a significant amount of albumin on day 1 (141.1 ± 21.3 ng/ml). However the amount was only slightly increased by day 3, then the albumin concentration decreased sequentially over the following 4 days. We speculated that the change in albumin concentration resulted from apoptosis of isolated hepatocytes and degeneration of the albumin protein itself. Urea production was also confirmed after 7-day culture of iPS-Heps in the bioreactor modules, suggesting the detoxication potential of ammonia via the urea cycle. However the amount of urea produced by iPS-Heps (0.1 ± 0.1 mg/dl) was much smaller than that produced by hepatocytes (0.6 ± 0.1 mg/dl).

Cell clusters attached to the extracapillary membrane were observed by scanning electron microscope (Figure 4C,D,E). These results indicated that iPS-Heps existed in the extracapillary space in an adhesive state, and those cells survived for up to 7 days of culture. Moreover, maturation of iPS cells into albumin-producing cells could be carried out in hollow fiber modules.

## Discussion

Transplant rejection, which can lead to early graft damage and loss, is a major problem in the field of transplantation medicine. Transplant rejection is caused by two mechanisms: antibody-mediated rejection and T cell-mediated rejection. Among these, antibody-mediated rejection is the more common cause of hyperacute rejection and chronic rejection. Pre-existing donor-specific antibodies can be generated by previous exposure to major histocompatibility antigens via blood transfusions, previous allografts or pregnancy. Such antibodies destroy transplanted tissue; therefore, patients present with immediate graft dysfunction [25,26]. On the other hand, *de novo* donor-specific antibodies that develop after transplantation are capable of triggering insidious graft injury in the late phase of post-transplantation. Protection against hyperacute rejection and chronic rejection are vital to BAL systems [27,28], as well as to organ transplantation and to cell transplantation [29].

Nyberg *et al.* tested a membrane with 0.2-μm pores and one with 400-kilodalton pores [10]. They observed more dead hepatocytes in the 0.2-μm pore group than in the 400-kilodalton pore group, with positive deposition of host IgG, IgM, and complements among the dead hepatocytes. Additionally, the cytotoxicity was reduced by heating the host serum for 30 min at 56°C, conditions known to denature the complement. Xhi *et al.* and Zhang *et al.* also reported similar results using a 200-kilodalton pore membrane and a 1200-kilodalton pore membrane [30,31]. Their results suggested that antibody-mediated immune responses involving complement systems occurred in a BAL system with a larger pore size. Accordingly, the use of a membrane with a smaller pore size has protective advantage from antibody-mediated rejection.

Another concern for BAL systems is the mass transfer efficiency of bioreactor devices. Principally, key functions of a BAL system, such as supply of synthesized protein to the host, detoxification, ureogenesis, ureagenesis and glucogenesis, are regulated by mass transfer through the membrane of the devices. Mass transfer rates are mainly determined by membrane permeability [24,30]. Larger pore size enables more efficient exchange of substances via the membrane of the module [28]. Conversely, a membrane with a large pore size may permit permeation of immunoglobulins that can cause antibody-mediated rejection of embedded hepatocytes.

In theory, a membrane with a smaller pore size than the molecular weight of immunoglobulins, e.g., IgG (147 kilodaltons), IgM (900 kilodaltons) and IgE (190 kilodaltons), completely shuts out permeation of immunoglobulins. In this study, IgG could not pass through a membrane with 100-kilodalton pores. This membrane pore size is larger than the molecular weight of representative substances metabolized by hepatocytes, such as albumin (67 kilodaltons), conjugated bilirubin (0.760 kilodalton), unconjugated bilirubin (0.585 kilodalton), and ammonia (0.017 kilodalton). However, albumin was not detected in the intracapillary flow even after circulation of medium for 180 min. Nedredal *et al.* reported similar results as ours: they investigated the mass transfer effect of hollow fiber modules with 70-, 150-, and 400-kilodalton pore sizes, and noted that albumin did not pass through the 70-kilodalton pore membrane, but did through the 150- and 400-kilodalton pore membranes [24]. We speculate that membrane fouling, due to albumin itself or materials in the circulating medium, caused the inhibition of albumin permeation. Nedredal *et al.* reported that a 70-kilodalton membrane showed a 62.5% reduction of permeability within 20 min after flushing of polydispersed dextran solution [24]. Lesser reduction rates were shown in the 150-kilodalton membrane (23.7%) and 400-kilodalton membrane (7.1%). These data indicate that exposure of a smaller-pore membrane to the medium or plasma can cause serious blockage of the pores. Thus, it seems impractical to construct an ideal BAL system, which would enable both efficient mass transfer and complete blockage of antibodies.

iPS cell-derived hepatocytes have great advantages over other cell sources for BAL systems, such as primary human hepatocytes, immortalized cell lines of C3A human hepatoblastoma cells, and cryopreserved porcine hepatocytes. Autotransplantation of hepatocytes differentiated from a patient’s own iPS cells is free from the issue of immunological rejection. Therefore, a membrane with the largest pore size can be employed for a BAL system with iPS cell-derived hepatocytes because blockage of immunoglobulins is not required. Obviously, the largest pore size for such a BAL system would allow a maximal mass transfer rate without membrane fouling. In this study, we applied hollow fiber modules with a 0.2-μm pore for iPS-Heps culture. iPS-Heps infused into the extracapillary space adhered to the surface of hollow fibers, and those cells were successfully cultured for 7 days. Consistent increase of the albumin concentration in the intracapillary flow indicated that iPS-Heps were functional during the culture period. In addition, albumin transfer via the membrane was operative for 7 days, without significant reduction of permeability. These data suggest that the combination of a 0.2-μm pore membrane and iPS cell-derived hepatocytes is a good candidate for an improved BAL system.

Unlimited proliferation is another advantage of iPS cells, whereas primary hepatocytes have knotty problems including limited supply, low viability after cryopreservation, prompt *in vitro* degeneration, and poor proliferative ability [2]. However, there are several obstacles to be overcome for iPS cell-oriented regenerative therapies. So far, differentiation of iPS cells into fully matured hepatocytes is hard to achieve. Even with modification of the differentiation protocol, complete differentiation of iPS cells *in vitro* has not yet been reported [8]. In a recent report, Yu *et al.* noted that iPS cell-derived hepatocytes also expressed high levels of alpha fetoprotein even by means of an improved protocol under a low oxygen condition, suggesting a persistent immature phenotype or an incapability to turn off early-stage genes [32]. Similarly, the final product of the differentiation program in this study showed much lower metabolic function compared with isolated hepatocytes. Thus, establishment of a differentiation protocol to generate fully matured hepatocytes should be given the highest priority to realize a BAL system with iPS cell-derived hepatocytes. A possible approach to achieve this goal is to differentiate iPS cells in an *in vivo* environment or coculture them with a combination of liver-populating cells [33]. High cost is another concern for iPS cell-oriented cell therapy, because expanding and differentiating iPS cells requires expensive reagents. Although such problems are still to be resolved before the clinical application of iPS cells, we believe that iPS cells should enable the production of patient-specific donor cells in the near future.

## Conclusions

In summary, we tested a BAL system embedded with iPS cells. iPS-Heps were injected into a module with a 0.2-μm pore membrane. Those cells adhered to the surface of hollow fibers and produced albumin and urea at least for 7 days. Although further investigation and improvement of both the devices and the differentiation program is required, we can conclude that the combination of a 0.2-μm pore membrane and iPS cell-derived hepatocytes shows promise for an improved BAL system. This paper provides the basic concept and preliminary data for an iPS cell-oriented BAL system.

## Competing interests

The authors declare that they have no competing interests.

## Authors’ contributions

MI carried out the cell culture and differentiation of iPS cells, PCR assay, ELISA assay and drafted the manuscript. HS participated in the design of the study. SN provided the bioreactor system and critically revised the manuscript for important intellectual content. MF performed transmission electron microscopy and scanning electron microscopy analysis. AT analyzed ELISA data and performed statistical analysis. NK and KY conceived the study, participated in its design and coordination, and helped to draft the manuscript. All authors read and approved the final manuscript.
